# A systematic review of studies describing the influence of informal social support on psychological wellbeing in people bereaved by sudden or violent causes of death

**DOI:** 10.1186/s12888-020-02639-4

**Published:** 2020-05-29

**Authors:** H. R. Scott, A. Pitman, P. Kozhuharova, B. Lloyd-Evans

**Affiliations:** 1 Division of Psychiatry, University College London, 6th floor, Maple House, 149 Tottenham Court Road, London, W1T 7BN UK; 2 Department of Psychology, University of Roehampton, London, UK

**Keywords:** Bereavement, Violent loss, Sudden loss, Social support

## Abstract

**Background:**

Whilst any type of bereavement can be traumatic, bereavement through violent or sudden causes is associated with more severe negative health and wellbeing outcomes compared to other types of loss. Social support has been found to have a positive impact on wellbeing after traumatic events in general. However, this association appears to be less consistently demonstrated in studies that focus on bereavement, and the literature in this area has not yet been systematically reviewed. This study aimed to review the international literature to examine systematically whether there is an association between informal social support from family and friends after bereavement through sudden and/or violent causes and post-bereavement wellbeing.

**Methods:**

We conducted a systematic search for quantitative studies that tested for an association between social support and any outcome related to wellbeing after a sudden and/or violent loss. Included studies were assessed for quality, and findings were reported using the approach of narrative synthesis. The review was pre-registered on Prospero (registration number CRD42018093704).

**Results:**

We identified 16 papers that met inclusion criteria, 11 of which we assessed as being of good or fair quality and 5 as poor quality. Fifteen different wellbeing outcomes were measured across all studies. We found consistent evidence for an inverse association between social support and symptoms/presence of depression, predominantly consistent evidence for an inverse association between social support and symptoms/presence of post-traumatic stress disorder (PTSD), and conflicting evidence for an inverse association between social support and symptoms/presence of complicated grief.

**Conclusions:**

Our systematic review identified evidence to suggest that social support after sudden or violent bereavement is associated with a reduced severity of depressive and PTSD symptoms. Further longitudinal research is needed to explore potential causality in this relationship, widening the focus from common mental disorders to include other mental illnesses, wellbeing outcomes, and suicide-related outcomes after bereavement. There is also a need for consensus on the conceptualisation and measurement of social support. Our findings imply that interventions to improve access to and quality of social support may reduce the burden of mental illness after bereavement, and may therefore be worth investing in.

## Background

Bereavement is a stressful life event that can have a long-lasting negative impact on wellbeing and quality of life [1]. All types of bereavement present a significant challenge in terms of adapting to life without the deceased. According to the dual process model [2], adapting to a loss requires dealing with both loss and restoration oriented stressors; dealing with the changes and feelings that relate to the death itself as well as the changes in roles and responsibilities it brings.

This model is compatible with the idea that certain types of loss are more challenging to adapt to than others [3]. Losses that are sudden (such as those arising from natural disasters, transport accidents) do not allow those left behind the chance to prepare: either for the loss of their relationship with the deceased or for any additional role they may take on, such as financial or caregiving duties. Violent losses (such as homicide or suicide) are also generally sudden, but are additionally challenging in terms of loss-oriented stressors as they violate the assumption that human life must be protected [4]. A systematic review [5] found consistent evidential support that losses that are both sudden and violent are distinct from other form of loss, being associated with slower recovery and an increased risk or prevalence of mental health disorders such as PTSD and depression compared to bereavement from natural deaths.

Social support has been proposed as protective against the negative effects of stressful life events [6]. Whilst the definition and conceptualisation of social support in research varies [7], informal social support describes the help provided by the individual’s existing social network, whereas formal social support describes organised help from individuals who may be professionals (such as trained peer group facilitators) or non-professionals (such as peer group members) [8]. There are two models through which this effect is proposed to work; the main effects model and the buffering model. The buffering model [9] suggests that social support has a protective effect on the negative impact of stressful life events by moderating the relationship between stress and wellbeing, rather than an overall positive effect on individuals regardless of their situation, as proposed in the main effects model [10]. In the wider literature, there is support for both models, but more consistent evidence for social support having an overall impact on wellbeing irrespective of levels of stress [11, 12]. The main effects model also takes into account the potential positive benefits of social support beyond negating stressors [13]. In particular, better social support is associated with a lower level of depressive and PTSD symptoms [6, 14].

There is limited empirical support for the effectiveness of formal social support interventions following sudden and violent loss [4], findings mirrored by evidence regarding those who have experience any kind of loss [15]. The same is true for more specific groups, such as those bereaved by suicide, where a recent systematic review has found that a diverse range of different interventions have been assessed for effectiveness using a range of outcomes measures, leading to inconclusive evidence for best practice [16]. Interventions based on peer support services, where individuals use shared experience to support one another, have a more consistent positive benefit [17]. However, formal sources of social support, including as peer support, must be sought out proactively. Research, however, shows that those bereaved by sudden causes are more likely to access informal social support [18], described as the provision of help from other people, typically emotional, tangible, informational and companionship support [19]. Informal social support is therefore the most accessable and personalisable type of support available to those bereaved through violent and/or sudden causes [20]: interventions to improve access to informal social support for people in this situation could therefore be valuable if its relationship to higher levels of wellbeing is established in this context.

The most recent review of the impact of informal social support on wellbeing outcomes after bereavement was carried out 14 years ago [21]. However, this was a non-systematic review that focussed on studies with a primary aim of testing the buffering hypothesis of social support but instead finding support for the main effects model. The eight included studies found mixed evidence to support social support after bereavement as having a significant impact on wellbeing. Given the specific nature of the inclusion criteria for interventions in that review, it is likely that a number of relevant papers examining the impact of social support after a loss were not included. Additionally, the mixed findings could be explained by the inclusion of heterogeneous samples bereaved by all types of loss.

To address an identified gap in current knowledge, our review sought to understand whether informal social support is associated with wellbeing after a loss through sudden and/or violent causes, by synthesising evidence from studies that compared measures of psychological wellbeing in those who received varying levels of informal support after bereavement.

## Methods

### Study inclusion

We included peer-reviewed primary observational (cross-sectional or longitudinal) research studies published as a full paper rather than solely an abstract, which used quantitative methods to investigate the association between social support and wellbeing of adults (18 years old or over) following bereavement through violent and/or sudden death. Violent deaths were defined as those that were unnatural and caused by human action [22], whereas sudden deaths were those that were unexpected and occurred instantly or rapidly [23]. It was a requirement that study participants identified as having had a personal relationship (friend or family member) with the deceased.

Exposure was defined as participants’ first-hand experience of any form of social support, provided by family or friends outside a formal setting (i.e. excluding peer mentoring groups or care-giving agencies) after their loss. We only included studies in which social support measures had been psychometrically validated regarding one or more of: content, criterion or construct validity. We included studies that assessed the outcomes of: i) psychological wellbeing, defined as positive psychological adjustment, measured using validated indicators of psychological adjustment (such as measures of social involvement, life satisfaction or sense of purpose); or ii) psychiatric symptoms (such as a clinical diagnosis of a mental health problem or a measure of mental health symptom severity assessed using a psychometrically validated assessment tool); or iii) a measure of service use in relation to mental health problems.

Our exclusion criteria were: studies that solely analysed data qualitatively or did not specify cause of death.

### Study selection

We registered the protocol for this review with PROSPERO: registration number CRD42018093704. Our search terms combined terms for: sudden or violent bereavement; and informal social support; and mental health or wellbeing (Additional file 1: Appendix 1). The protocol was reviewed by our Public and Patient Involvement (PPI) representatives who confirmed that the review question was of value and commented on the search terms, and also by a university librarian (see Acknowledgements).

We conducted a systematic search of five online databases: IBSS, CINAHL, PsychINFO, MEDLINE and the Cochrane library. Our inclusion criteria were observational studies published from database inception up to 26th April 2018 without language or date limits. The search was updated a year later, with records searched up to 10th May 2019.

In addition to the database searches, we hand-searched from journal inception three relevant journals, *Bereavement Care*, *Death Studies* and *OMEGA- The Journal of Death and Dying*. We also hand-searched conference abstracts from all available online records of key relevant conferences (International Death, Grief and Bereavement conference; European Symposium on Suicide and Suicidal Behaviour) as well as grey literature sources (OpenGrey, OpenDOAR, EThOS and OATD databases searched). For each study identified for inclusion in the review, we hand-searched the reference list and used forward citation tracking. We extracted and managed references using Endnote software.

For 29 studies that reported they had recorded death type but not distinguished between types of death in statistical analyses, authors were contacted to request further information.

To screen references we followed the Preferred Reporting Items for Systematic Reviews and Meta-Analyses (PRISMA) guidelines ([24]; checklist included as Additional file 1: appendix 2). The first author reviewed all titles, identifying abstracts for review, and thereby full text articles for review. A second author (PK) independently reviewed 15% of article abstracts and full text articles, with any disagreements discussed between authors.

### Data extraction

We developed a standardised schedule to extract data (attached as Additional file 1: appendix 3) and summarise details of the study setting, sample, measures of intervention and outcome and results. The second author independently extracted data from 15% of the included papers, with any disagreements discussed between authors.

### Quality appraisal

Following data extraction, we used the Newcastle-Ottawa Scale for evaluating the quality of non-randomised studies [NOS; [22]] to assess the quality of the included longitudinal studies three domains: selection, comparability and outcome. Discounting the criteria covering the selection of a non-exposed cohort that would not be applicable to single-group studies, a maximum score of 8 was possible. As the NOS has been designed primarily for cohort and case control studies, an adapted version of the NOS [25] was used to assess the quality of the included cross-sectional studies. A maximum score of 10 was possible for this scale.

The first and second reviewing authors independently reviewed each of the included studies according to the criteria set out in the tool, and where disagreements arose over assessment of bias, these were discussed with other authors.

We interpreted scores according to the rating system used for the standard NOS. To be rated as being good quality, studies had to score 3 or 4 points in the selection domain, 1 or 2 points in the comparability domain and 2 or 3 points in the outcome/exposure domain. For fair quality, studies had to score 2 points in selection domain, 1 or 2 points in the comparability domain and 2 or 3 points in the outcome/exposure domain. Studies were deemed to be poor quality if they scored 0 or 1 point in the selection domain, 0 points in comparability domain or 0 or 1 points in the outcome/exposure domain.

### Analysis

As we expected that included studies would be heterogeneous in terms of conceptualisations of social support, study settings, participant characteristics and the measures and statistical analyses used, we did not anticipate conducting a meta-analysis but instead planned to use the approach of narrative synthesis, grouping findings by outcome. For this we referred to an existing framework [26] to ensure a systematic approach. This framework starts by developing a theory of how the exposure works, why and for whom, before developing a preliminary synthesis of findings, exploring relationships in the data, and assessing robustness of the synthesis. When discussing study results, we used “positive association” if all measured social support variables had a significant positive association with the reduced severity of, or reduced likelihood for meeting the threshold of diagnosis for a measured outcome. We used “partial positive association” if some but not all of the included social support variables had a significant positive association with reduced severity of, or reduced likelihood for meeting the threshold of diagnosis for the measured outcome, and the remaining included variables were not significantly associated with the outcome.

It was planned that results would initially be grouped by outcome, with specific sub-group analysis based on type of loss or social support measurement reported where appropriate.

## Results

### Included studies

**Fig. 1:**
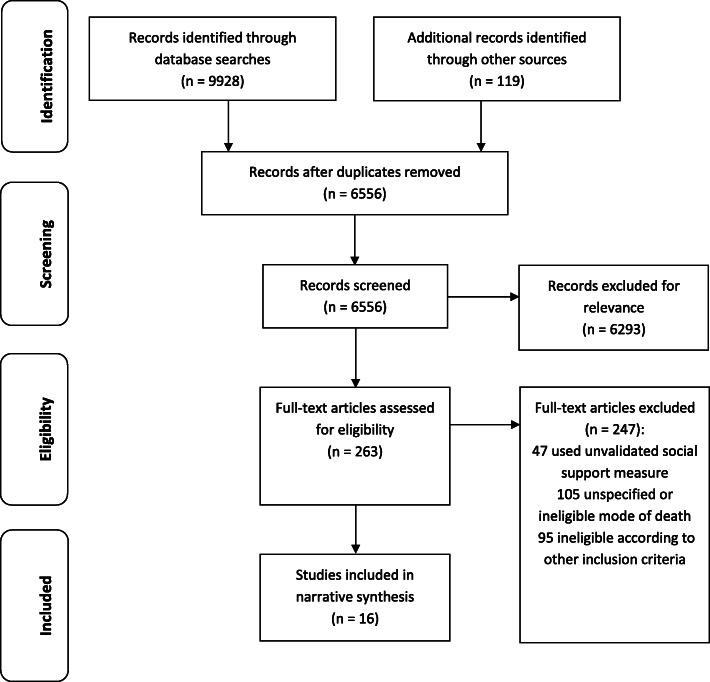
Flow diagram of included studies

Using electronic database searches we identified 6556 records for title and abstract screening after removing duplicates (Fig. 1). We conducted a full text review of 263 records, of which 16 met all the inclusion criteria and were included in the narrative synthesis. Foreign language full text articles were translated (seven in Japanese, two in Spanish, two in German, two in Chinese (simplified) and one in French). No additional studies were found through grey literature searching, or hand searches of journal contents of included studies’ reference lists.

Initial rates of agreement between the two reviewing authors were 97% for screening, 98% for data extraction and 98% for the quality assessment. All disagreements were resolved through discussion.

### Study characteristics

The 16 included papers reported results from 15 different studies, with one study reported in two included papers [27, 28] at different follow-up time points.

Of the 15 samples included (Table 1), nine sampled populations in North America (USA and Canada) [27–29, 31, 33, 45, 47, 48, 50], two in China [44, 52] two in Israel [41, 42], one in Colombia [38] and one in Norway [40]. The earliest study was published in 1985 and the most recent in 2019. The sample size of included studies ranged between 44 and 803 participants. Mean age of samples ranged between 33 and 79 and, except for one study, the majority of participants in each sample were female. Participant groups were defined as those bereaved by natural disasters [27, 40, 44, 52, 54], homicide [29, 31, 33, 47], suicide [42, 43, 45, 48], accidental death [36, 50] or armed conflict [38]. One study was longitudinal in design [26], and measured outcomes 6 months after baseline measurement (at a mean of 1.66 years post-loss). Another study [55] followed-up a sample described in an included cross-sectional analysis [27] but reported different measures, so was essentially a separate cross-sectional analysis and not comparable. All other studies were cross-sectional in design.

**Table 1:** Study characteristics

Author, year, country	Study design Sample source	Sample demographics	Mode of bereavement Time since bereavement	Social support measures	Measured outcomes
Bailey, 2013, Canada [29]	Cross-sectional Community organisation	*n*=48 mean age=51.5 100% female	Child lost to gun violence 0.5-12 years	Multidimensional Scale of Perceived Social Support (MSPSS) [30]	Resilience
Bottomley, 2017, U.S.A. [31]	Longitudinal (6 month follow-up) Support organisation	*n*=47 mean age=49.7 89.4% female	Family member lost to homicide Mean length= 1.66 years at T1, 2.16 at T2	Arizona Social Support Interview Schedule (ASSIS) [32]	PTSD, complicated grief, depression, anxiety
Burke, 2010, U.S.A. [33]	Cross-sectional Support organisation	*n*=54 mean age=48.6 88.9% female	Family member lost to homicide Mean length= 1.75 years	ASSIS, Inventory of Social Support (ISS) [34] & MSPSS	PTSD, complicated grief, depression
Cowan, 1985, U.S.A.^a^ [27]	Cross-sectional Death certificates/ court records	*n*=119 (50 control) mean age=unclear 70% female	Friends and family lost in natural disaster Mean length= .92 years	Coppel Index of Social Support (CISS) [35]	Depression
Murphy, 1988, U.S.A.^a^ [28]	Cross-sectional Death certificates/ court records Official population records	*n*= 49 (bereaved) /36 (control) mean age= 30/37 74%/65% female	Friends and family lost in natural disaster Mean length= 3 years (estimate)	CISS	Mental distress, recovery
Fullerton, 1999, U.S.A. [36]	Cross-sectional Air force squadron	*n*=71 mean age=33 4.0% female	Squadron members of personnel lost in plane crash Mean length= 0.17 years	Perceived Social Support Scales (Family and Friends) [37]	Depression, initial impact of event
Heeke, 2017, Colombia [38]	Cross-sectional Humanitarian organisation	*n*=308 mean age=48.5 61.7% female	Significant other lost in armed conflict Mean length= 12.4 years	DUKE-UNC Functional Social Support Questionnaire [39]	PTSD, Prolonged grief, emotional distress
Kristensen, 2010, Norway [40]	Cross-sectional Official population records (police deceased list and population register)	*n*=130 mean age=45.7 51.5% female	Family member lost in natural disaster Mean length= 2.2 years	Crisis Support Scale (CSS) [41]	Complicated grief
Levi-Belz, 2015, Israel [42]	Cross-sectional Support organisation/online support forum	*n*=135 mean age=40.3 77.0% female	Family member lost to suicide Mean length = 3.5 years	MSPSS	Stress-related growth
Levi-Belz, 2019, Israel [43]	Cross-sectional Support organisation/online support forum/online advertising	*n*=156 mean age=40.7 81.4% female	Family member or friend lost to suicide Mean length = 10 years	MSPSS	Complicated grief
Li, 2015, China [44]	Cross-sectional Official population records	*n*=803 mean age=46.7 63% female	Family lost to natural disaster Mean length = 1.0 years	MSPSS	Complicated grief
Oexle, 2019, U.S.A [45]	Cross-sectional Support organisation/online advertising	*n*=195 mean age=50 92% female	Immediate family lost to suicide Mean length = 8.9 years	Perceived Social Support Questionnaire (PSSQ) [46]	Depression, personal growth, grief difficulties, suicidal ideation
Rheingold, 2015, U.S.A. [47]	Cross-sectional Official population records	*n*=47 mean age=78.7 78.7% female	Immediate family lost to homicide Mean length = 2.1 years	ISS	PTSD, complicated grief, depression
Spino, 2016, U.S.A. [48]	Cross-sectional Social support group/online advertising	*n*=44 mean age=44 75% female	Adults bereaved by suicide Length of loss unclear	Norbeck Social Support Questionnaire (NSSQ) [49]	Depression, loneliness
Sprang, 1998, U.S.A. [50]	Cross-sectional Support organisation	*n*=171 mean age=34 54.4% female	Immediate family killed by drunk driver Mean length = 2.3 years	Provisions of Social Relations Scale (PSRS) [51]	PTSD, grief, mourning
Xu, 2017, China [52]	Cross-sectional Official population records	*n*=176 mean age=54.7 52.3% female	Child lost to natural disaster 6.0-6.3 years	Social Support Rating Scale (SSRS) [53]	PTSD

^a^Murphy [28] is a follow-up of Cowan [27], but measured different outcomes so is not comparable

Across the 15 different studies, 11 different validated measures of social support were used (Table 2). The Multidimensional Scale of Perceived Social Support (MSPSS) [57] was the most frequently included measure, employed in five studies [29, 33, 42–44].

**Table 2:** Social support measures used in studies included in this review

Measure	Type of social support assessed by measure	Type of measurement tool	Use of measure in included study	
Arizona Social Support Interview Schedule (ASSIS) [32]	Size and availability of and satisfaction with support network.	Structured interview.	Bottomley 2017 [31]	12 variables derived. Perceived need for and satisfaction with each of 6 categories: intimate interaction, material aid, advice and information, positive feedback, physical assistance, social participation
			Burke 2010 [33]	5 variables derived: available support network for family and non-family, actual support network, anticipated and actual negative relationships
Coppel Index of Social Support (CISS) [35]	Structural and functional support	Self-report questionnaire. 15 items on a 5 point Likert scale	Cowan 1985 [27]	Items across domains summed for total score of perceived social support.
			Murphy 1988 [28]	Items across domains summed for total score of perceived social support.
Crisis Support Scale (CSS) [41, 56]^a^	Received social support	Self-report questionnaire. 7 items on a 7 point Likert scale	Kristensen 2010 [40]	Scandinavian version. Factors summed separately to measure positive social support and negative social response.
DUKE-UNC Functional Social Support Questionnaire [39]	Functional social support	Self-report questionnaire. 11 items on a 5 point Likert scale	Heeke 2017 [38]	Translated version. Items summed for total score of perceived social support.
Inventory of Social Support (ISS) [34]^a^	Perceived social support for grief	Self-report questionnaire. 5 items on a 5 point Likert scale	Burke 2010 [33]	Items summed for total score of available grief support.
			Rheingold 2015 [47]	Items summed for total score of perceived social support.
Multidimensional Scale of Perceived Social Support (MSPSS) [57]	Perceived presence and level of support across three domains: family, friends and significant other.	Self-report questionnaire. 12 items on 7 point Likert scale	Bailey 2013 [29]	Items across domains summed for total score of perceived social support.
			Burke 2010 [33]	Items across domains summed for total score of available general support.
			Levi-Belz 2015 [42]	Items across domains summed for total score of available perceived support.
			Levi-Belz 2019 [43]	Items across domains summed for total score of perceived support.
			Li 2015 [44]	Translated version. Items across domains summed for total score of general social support.
Norbeck Social Support Questionnaire (NSSQ) [49, 58]	Perceived social support: functional support	Self-report questionnaire. Amount of support from supportive network members listed.	Spino 2016 [48]	Network score, relationship score and both combined for total score.
Perceived Social Support Scales, friends and family (PSS-Fr, PSS-Fa) [37]	Perceived social support from friends and family	Self-report questionnaires. 20 items on a 3 point Likert scale	Fullerton 1999 [36]	Items summed for each scale for total score of support from friends and support from family.
Provisions of Social Relations Scale (PSRS) [51]	Perceived social support	Self-report questionnaire. 18 items on a 5 point Likert scale	Sprang 1998 [50]	Family support and friend support subscales combined for a total score of cognitive appraisal of support.
Perceived Social Support Questionnaire (PSSQ) [46]	Perceived social support	Self-report questionnaire. 6 items on a 5 point Likert scale	Oexle 2019 [45]	Items summed for a total score of perceived support.
Social Support Rating Scale (SSRS) [53]	Subjective support, objective support and support availability	Self-report questionnaire developed for Chinese populations. 10 items	Xu 2017 [52]	Three domains of social support combined for a total score and categorised into low, medium and high support.

^a^Assessment tools that have 2 references by their name are those that have been initially described in one study and validated in a separate study. All other assessment tool references include an initial description and validation of the tool in one study

Measures were based on different theoretical approaches to social support, with some distinguishing between perceived and received social support (measuring one or both), and some distinguishing between structural support (integration with social network) and functional support (specific functions provided by others) and measuring one or both [59], and some developed and validated for specific populations.

Across the 15 different studies, 15 different mental health and psychological wellbeing outcomes were measured. The most frequently measured outcomes were post-traumatic stress disorder [31, 33, 38, 47, 50, 52], depression [27, 31, 33, 36, 45, 47, 48] and complicated grief [31, 33, 40, 43, 47]. The remaining measures were of other distinct psychiatric and psychological wellbeing outcomes (Table 3). No studies measured service use as an indicator of wellbeing. Where studies measured prevalence of an outcome rather than symptom severity, a cut-off score on an assessment tool was used rather than self-report of an existing clinical diagnosis.

**Table 3:** Findings grouped by outcome

Outcome	Study	Exploratory or specific hypothesis	Analysis method	Covariates included in models	Sample size (n)	Findings
**Psychiatric outcomes**						
PTSD	Bottomley 2017 [31]	Exploratory	Regression model with social support as a predictor.	T1 PTSD (at a mean of 1.66 years since loss)	47	Of 12 social support variables, need for advice, need for physical assistance and satisfaction with physical assistance were included in the model. Satisfaction with physical assistance was the only significant predictor, negatively predicting PTSD severity at T2 (6 month follow-up) (*p*<.03, b=-.18).
	Burke 2010 [33]	Exploratory	Correlations	n/a	54	Of 6 variables measured, percentage of actual negative relationships significantly correlated with PTSD severity (.28, *p*<.05).
	Heeke 2017 [38]	Specific hypothesis	Latent class analysis	Gender, years of education, number of assaultive/accidental traumatic events, relationship to person lost, how loss happened and time since loss.	308	Social support was the only factor associated with PTSD symptoms compared to the resilient class (OR= .95, *p*=.005).
	Rheingold 2015 [47]	Exploratory	Generalised estimating equations	Variables found to significantly differ by diagnostic status: employment status, deceased contributing to household income.	47	Lack of social support was independently associated with increased risk of meeting criteria for PTSD (beta =.19, Wald x2 = 4.64, *p*<.05).
	Sprang 1998 [50]	Exploratory	Regression model with social support as a predictor.	Gender, age, race, subjective health status, income, marital status, past experience with death, time since death and religious beliefs.	171	Greater social support was associated with lower rates of PTSD symptoms (beta=.415, *p*<.005; 43.2% of variance).
	Xu 2017 [52]	Exploratory	Regression model with social support as a predictor.	Ethnicity, residence location, gender, age, monthly income, education level, age of child and gender of child.	176	Low social support was a significant risk factor for meeting criteria for PTSD (OR= .244, beta=-1.41, *p*=.002, 95% CI).
Depression	Bottomley 2017 [31]	Exploratory	Regression model with social support as a predictor.	T1 depression (at a mean of 1.66 years since loss)	47	Of twelve social support variables, need for advice, need for physical assistance and satisfaction with physical assistance were included in the model but none were significant predictors.
	Burke 2010 [33]	Exploratory	Correlations	n/a	54	Of six social support measures, two were significantly correlated with depression severity: grief support (-.27, p<.05) and percentage of anticipated negative relationships (.28, *p*<.05).
	Cowan 1985 [27]	Exploratory	Regression model with social support as a predictor.	Stress, age, gender, importance of deceased and perceived preventability of death.	69	Perceived social support was associated with greater depression severity (*p*<.05, b=-.14), accounting for 38% of variance in the model.
	Fullerton 1999 [36]	Exploratory	Regression model with social support as a predictor.	Age, marital status, social network index, disaster specific social support, family distress, maximum closeness to deceased crew, transience, hardiness, social desirability and initial impact of event (IES).	71	Support from friends and support from family were entered as separate predictors in each model. In models controlling for total IES and IES intrusion scores, neither perceived social support variable was associated with depression severity. Controlling for IES avoidance (10%), perceived social support from friends was negatively associated with depression severity (5% of variance; beta=-.03, *p*=.027).
	Oexle 2019 [45]	Specific hypothesis	Regression model with social support as a predictor.	Age, gender, pre-loss mental illness, time since loss, relationship to deceased and perceived closeness to deceased.	195	Greater perceived social support was significantly associated with a lower level of depressive symptoms (beta=-.53, *p*<.001).
	Rheingold 2015 [47]	Exploratory	Generalised estimating equations with social support as a predictor.	Variables found to significantly differ by diagnostic status: age, employment status, deceased contributing to household income.	47	Lack of social support was independently associated with increased risk of meeting criteria for MDD (beta =.40, Wald x2 = 14.37, *p*<.005).
	Spino 2016 [48]	Specific hypothesis	Regression model with social support as a predictor.	Physical health encumbrance.	44	Three social support variables were used as predictors. In a linear regression model, higher network score was associated with a significant decrease depression severity (beta= -0.53, *p*=.011). In a linear regression model, higher relationship score was associated with a significant decrease depression severity (beta= -0.18, *p*=.011). In the multiple regression model, higher total support score (beta= -0.02, *p*=.001) was associated with a significant decrease in depression severity.
Complicated grief	Bottomley 2017 [31]	Exploratory	Regression model with social support as a predictor.	T1 complicated grief (at a mean of 1.66 years since loss)	47	Of twelve social support variables, satisfaction with physical assistance was the only significant predictor out of the three social support variables included in the model, positively predicting complicated grief severity at T2 (6 month follow-up) (beta=.20, *p*<.05).
	Burke 2010 [33]	Exploratory	Correlations	n/a	54	Of six social support measures, two were significantly correlated with complicated grief severity: percentage of actual negative relationships (.28, *p*<.05) and available support system (-.28, *p*<.05).
	Kristensen 2010 [40]	Exploratory	Regression model with social support as a predictor.	Gender, pre-disaster employment, relationship to deceased, previous experience of loss, time elapsed before death confirmed.	130	Two social support variables were included in analysis: low positive social support (OR=.24, *p*=.012) and high negative social support (OR=3.81, *p*=.012) were significantly associated with meeting criteria for complicated grief.
	Levi-Belz 2019 [43]	Specific hypothesis	Regression model with social support as a predictor.	Time since loss, attachment style, self-disclosure and interaction between secure attachment, social support and self-disclosure.	156	Greater perceived social support was significantly associated with lower severity of complicated grief (beta=-.30, *p*<.01).
	Li 2015 [44]	Exploratory	Regression model with social support as a predictor.	n/a	803	Social support was not significantly associated with meeting criteria for complicated grief.
	Rheingold 2015 [47]	Exploratory	Generalised estimating equations with social support as a predictor.	Variables found to significantly differ by diagnostic status: age, deceased contributing to household income.	47	Lack of social support was not significantly associated with increased risk of meeting criteria for complicated grief.
Anxiety	Bottomley 2017 [31]	Exploratory	Regression model with social support as a predictor.	T1 anxiety (at a mean of 1.66 years since loss)	47	Need for advice, need for physical assistance and satisfaction with physical assistance were included in the model. Satisfaction with physical assistance was the only significant predictor, negatively predicting anxiety severity at T2 (6 month follow-up) (*p*<.001, b=-.30).
Prolonged grief	Heeke 2017 [38]	Specific hypothesis	Latent class analysis with social support as a predictor.	Gender, years of education, number of assaultive/accidental traumatic events, relationship to person lost, how loss happened and time since loss.	308	The amount of perceived social support did not predict membership of the PGD class.
Suicidal ideation	Oexle 2019 [45]	Specific hypothesis	Regression model with social support as a predictor.	Age, gender, pre-loss mental illness, time since loss, relationship to deceased and perceived closeness to deceased.	195	Greater perceived social support was significantly associated with lower severity of suicidal ideation (beta=-2.87, *p*<.001).
**Psychological wellbeing outcomes**						
Emotional distress	Heeke 2017 [38]	Specific hypothesis	Latent class analysis with social support as a predictor.	Gender, years of education, number of assaultive/accidental traumatic events, relationship to person lost, how loss happened and time since loss.	308	Less social support was a predictor of the emotional distress class (OR= .92, *p*<.001).
Grief	Sprang 1998 [50]	Exploratory	Regression model with social support as a predictor.	Gender, age, race, subjective health status, income, marital status, past experience with death, time since death and religious beliefs.	171	Greater social support predicted lower extent of grief (beta=-.479, *p*<.005).
Grief difficulties	Oexle 2019 [45]	Specific hypothesis	Regression model with social support as a predictor.	Age, gender, pre-loss mental illness, time since loss, relationship to deceased and perceived closeness to deceased.	195	Greater perceived social support was significantly associated with decreased grief difficulties (beta=-.47, *p*<.001).
Initial impact of event	Fullerton 1999 [36]	Exploratory	Regression model with social support as a predictor.	Age, marital status, social network index, disaster specific social support, family distress, maximum closeness to deceased crew, transience, hardiness and social desirability.	71	Neither perceived social support measure (support from friends/ support from family) was a good predictor of total or avoidance IES. Low perceived social support from friends predicted a higher intrusive initial IES score (beta=-.44, *p*=.044).
Loneliness	Spino 2016 [48]		n/a	n/a	n/a	Statistical analyses not reported.
Mental distress	Murphy 1988 [28]	Exploratory	Regression model with social support as a predictor.	T1 mental distress, age, sex, education, stress, self-efficacy and social support	49	Social support did not significantly predict severity of mental distress
Mourning	Sprang 1998 [50]	Exploratory	Regression model with social support as a predictor.	Gender, age, race, subjective health status, income, marital status, past experience with death, time since death and religious beliefs.	171	Greater social support significantly predicted lower extent of mourning (beta=.350, *p*<.005).
Personal growth	Oexle 2019 [45]		Regression model with social support as a predictor.	Age, gender, pre-loss mental illness, time since loss, relationship to deceased and perceived closeness to deceased.	195	Greater perceived social support was significantly associated with increased personal growth (beta=-44, *p*<.05).
Recovery	Murphy 1988 [28]	Exploratory	n/a	n/a	n/a	Social support was not included in the regression model predicting recovery.
Resilience^a^	Bailey 2013 [29]	Exploratory	Regression model with social support as a predictor.	n/a	48	Unadjusted model where traumatic stress predicted greater levels of resilience was significant (b = -.241, p<.049). The adjusted model with social support as a mediator was also significant (b=.297, *p*=.032).
Stress-related growth	Levi-Belz 2015 [42]	Specific hypothesis	Regression model with social support as a predictor.	Time since loss, adaptive coping, maladaptive coping, self-disclosure, interaction between time and interpersonal variables.	135	Combined with self-disclosure as a predictive interpersonal variable, social support predicted levels stress-related growth (beta=.11, *p*=.027).

Key: ^a^Resilience was defined as stress coping ability

### Quality assessments

Table 4 shows the results of the NOS quality assessments for included studies. Most studies were judged as either good quality [40, 43, 45, 47, 50, 52] or fair quality [27, 28, 31, 36, 38], and five studies were rated as poor quality [29, 33, 42, 44, 48]. The most frequent source of bias was sample size. No studies were deemed to have a justified sample size as none had carried out a power calculation. Low response rate or no response rate, and lack of comparison between respondents and non-respondents were also a common source of bias across studies, where 13 studies did not meet the criteria to score a point in this category.

**Table 4:** Newcastle-Ottawa Quality Assessment Scale

Adapted for cross-sectional studies									
	Selection				Comparability	Outcome			
Study	Representativeness of sample	Sample size	Non-respondents	Ascertainment of exposure	Confounding factors controlled	Assessment of the outcome	Statistical test		Quality
Bailey 2013 [29]	1	0	1	2	0	1	1		Poor
Burke 2010 [33]	0	0	0	2	0	1	1		Poor
Cowan 1985 [27]^a^	1	0	0	2	1	1	1		Fair
Murphy 1988 [28]	1	0	0	2	1	1	1		Fair
Fullerton 1999 [36]	1	0	0	2	1	1	1		Fair
Heeke 2017 [38]	0	0	0	2	2	1	1		Fair
Kristensen 2010 [40]	1	0	1	2	1	1	1		Good
Levi-Belz 2015 [42]	1	0	0	2	0	1	1		Poor
Levi-Belz 2019 [43]	1	0	0	2	2	1	1		Good
Li 2015 [44]	1	0	0	2	0	1	1		Poor
Oexle 2019 [45]	0	0	1	2	2	1	1		Good
Rheingold 2015 [47]	1	0	0	2	1	1	1		Good
Spino 2016 [48]	0	0	0	2	0	1	1		Poor
Sprang 1998 [50]	1	0	0	2	2	1	1		Good
Xu 2017 [52]	1	0	0	2	1	1	1		Good
NOS for cohort studies									
	Selection				Comparability	Outcome			
	Representativeness	Selection of non-exposed cohort	Ascertainment of exposure	Outcome of interest not present at start of study	Comparability of cohorts	Assessment of outcome	Follow-up long enough for outcome to occur	Adequacy of follow-up	Quality
Bottomley 2017 [31]	0	n/a	1	0	1	1	1	1	Fair

^a^taking into account only participants who were bereaved, not control participants

In addition to the NOS, we noted that exploratory approaches were common, with multiple statistical models often used in study analyses, reflecting multiple outcomes and exposure variables. There was also great variation in the degree to which analyses controlled for potential confounding variables, and in the specific variables chosen as potential confounders, resulting in a risk of residual confounding in reported estimates.

### Summary of findings

Table 5 summarises the overall findings extracted from included studies for each outcome type.

**Table 5:** Summary of the number of studies indicating an association between social support and each outcome

	Number of studies indicating an association between social support and outcome			
	Positive association^a^	Partial positive association^b^	No association	Negative association
**Outcome**				
Psychiatric				
Depression (N = 7)	4 [27, 45, 47, 48]	2 [33, 36]	1 [31]	–
PTSD (*N* = 6)	4 [38, 47, 50, 52]	2 [31, 33]	–	–
Complicated grief (N = 6)	2 [40, 43]	1 [33]	2 [44, 47]	1 [31]
Prolonged grief (N = 1)	–	–	1 [38]	–
Anxiety (N = 1)	–	1 [31]	–	–
Suicidal ideation (N = 1)	1 [45]	–	–	–
Psychological				
Emotional distress (N = 1)	1 [38]	–	–	–
Grief (N = 1)	1 [50]	–	–	–
Grief difficulties (N = 1)	1 [45]	–	–	–
Initial impact of event (N = 1)	–	1 [36]	–	–
Mental distress (*N* = 1)	–	–	1 [28]	–
Mourning (*N* = 1)	1 [50]	–	–	–
Personal growth (N = 1)	1 [45]	–	–	–
Resilience (N = 1)	1 [29]	–	–	–
Stress-related growth (N = 1)	1 [42]	–	–	–

^a^ all measured social support variables had a significant positive association with the reduced severity of, or reduced likelihood for meeting the threshold of diagnosis for a measured outcome

^b^ some but not all of the included social support variables had a significant positive association with reduced severity of, or reduced likelihood for meeting the threshold of diagnosis for the measured outcome, with the remaining included variables not significantly associated with the outcome

#### Psychiatric outcomes

##### Depression (seven studies)

There was limited evidence that social support was associated with reduced risk of meeting the threshold for depression diagnosis or reduced depression symptom severity, with seven studies [27, 31, 33, 36, 45, 47, 48] measuring this outcome. The single longitudinal study [31] included in this review was of fair quality and was exploratory in nature, but did control for baseline outcome measures. This study found no association between the two variables.

Four studies [27, 45, 47, 48] reported a positive association between measures of social support and depression; two were good quality [45, 47], one was fair quality [27] and one was poor quality [48].

Two more exploratory studies reported a partial positive association between social support and depression. A study judged as fair quality [36] found that only one (perceived support from friends) of two social support variables in one of three analysis models was cross-sectionally associated with reduced symptom severity, with the other 2 models finding no association. A poor quality study [33] found that two (grief support and percentage of anticipated negative relationships) of six social support variables correlated significantly with reduced symptom severity.

##### PTSD (six studies)

There was limited evidence that social support was associated with a reduced risk of meeting the threshold for PTSD diagnosis or with reduced symptom severity. All six studies [31, 33, 38, 47, 50, 52] that measured PTSD as an outcome found some evidence of an association between increased social support and reduced severity of/likelihood of meeting threshold for PTSD, however studies were of mixed quality.

In the longitudinal study [31], one (satisfaction with physical assistance) out of 12 measured social support variables predicted lower symptom severity. Another poor quality study [33] found a partial positive association, with only one (percentage of actual negative relationships) of out six social support variables correlated with lower symptom severity.

Four other studies [38, 47, 50, 52] found a positive association between social support and PTSD. Three of these studies were of good quality [47, 50, 52] and one was of fair quality [38].

##### Complicated grief [CG] (six studies)

There was mixed evidence regarding whether social support was associated with a reduced risk of meeting the threshold for CG diagnosis or reduced symptom severity, with six studies [31, 33, 40, 43, 44, 47] measuring this outcome. The included longitudinal study [31] found that only one (satisfaction with physical assistance) of 12 social support variables was associated with CG, predicting increased severity of symptoms.

Two studies reported a positive association: two good quality studies [40, 43] reported a positive association between the social support risk of CG. Another study [33] found a partial positive association; this poor quality study found that two (percentage of actual negative relationships and available support system) of six social support variables was correlated with reduced symptom severity of CG.

Two more studies [44, 47], one poor quality [44] and one good quality [47], found no cross-sectional association between social support and CG.

In one fair quality cross-sectional study [38] assessed the outcome of prolonged grief, a concept similar to CG, and found no association with social support.

#### Other psychiatric outcomes (two studies)

The outcome of anxiety was measured in the included longitudinal study [31], where one of 12 measured social support variables at T1 significantly predicted lower levels of anxiety at T2 and the other variables showing no association.

A separate good quality study [45] found a significant positive association between a global social support measure and lower levels of suicidal ideation.

##### Other psychological wellbeing outcomes (eight studies)

Nine separate psychological wellbeing outcomes were measured, demonstrating limited evidence that social support is associated with improved psychological wellbeing.

There was consistent evidence that social support influences positive wellbeing, with three separate studies [29, 42, 45] measuring personal growth, stress-related growth and resilience. A good quality study [45] found that increased personal growth was cross-sectionally associated with increased social support, and a low quality study [42] found that increased stress-related growth was cross-sectionally associated with increased social support. Social support mediated the association between traumatic stress and resilience in a poor quality study [29].

The similar constructs of grief, mourning, and extent of grief difficulties, were each significantly cross-sectionally associated with social support in two separate exploratory studies [45, 50], both high quality.

Two studies measured distress with conflicting findings; one fair quality study [38] found a positive association between social support and emotional distress whereas another fair quality study [28] found no cross-sectional association between social support and mental distress.

A single fair quality study [36] assessed the initial impact of event (IES) and found that one (perceived support from friends) of two social support variables in one of three analysis models was cross-sectionally associated with reduced impact, the other two models finding no association.

Two further psychological outcomes, loneliness [48] and recovery [28], were mentioned as having been measured in the methods sections of separate studies but were not included in statistical analysis models reported.

#### Subgroup: people bereaved by suicide (four studies)

Four of the cross-sectional studies reported above [42, 43, 45, 48] included only participants who had been bereaved by suicide, each controlling for a range of demographic and health-related variables. Study results consistently found that increased social support was associated with improved wellbeing.

One poor quality study [42] found a partial positive association between social support and stress-related growth, and another good quality study [43] found that social support was cross-sectionally associated with a significantly reduced risk of CG.

Two other exploratory cross-sectional studies [45, 48], one good quality [45] and one poor quality [48], demonstrated a positive association between social support and depressive symptoms, suicidal ideation and grief difficulties.

#### Other subgroups

No other meaningful patterns of results defined by subgroups became apparent during the process of data synthesis, whether based on type of loss or type of social support measurement. Insufficient information was provided in studies to compare results by relationship type or time since loss and the limited number of longitudinal studies did not allow for consideration of whether studies support or refute the main effects or buffering models of social support.

## Discussion

### Main findings

To the authors’ knowledge, this is the first systematic review of studies describing the relationship between post-loss social support and psychological wellbeing after sudden and/or violent bereavement. We found only one longitudinal study among a total of 16 identified observational studies. From these studies, we found limited yet consistent evidence that receipt of greater social support is associated with lower severity/risk of PTSD, and that social support is associated with better psychological wellbeing after bereavement by suicide. We found predominantly consistent evidence that social support is associated with lower severity of depressive symptoms/risk of depression, but a longitudinal study found no association. We found conflicting evidence for an association between social support and CG severity/risk. For the majority of other psychiatric and psychological wellbeing outcomes measured in this body of literature, apart from mental distress, each was associated with social support, but for each this was only assessed in a single study.

On balance, the evidence suggests that better social support after sudden or violent bereavement is associated with better psychological wellbeing, and that this is a consistent finding among those bereaved by suicide. However, there are a number of key limitations of the current body of literature, as highlighted throughout this review, and summarised below. This suggests a need for high quality cohort studies to test this hypothesis further.

### Strengths and limitations of included studies

The tendency of included studies to focus on three clinical outcomes of PTSD, depression and CG mirrors that of other reviews measuring these outcomes [60–62], generally finding these to be more common or severe among people who experience and traumatic losses as compared to other bereavements. There is clearly a need to measure other outcomes post-bereavement, including substance use, suicide attempt, and severe mental illness, as well as non-clinical outcomes such as blame, guilt and emptiness [63]. However, one explanation for this is that validated measures for psychiatric outcomes are more available than those for non-clinical constructs.

We found similar methodological weaknesses in a number of the included studies; notably the use of small sample sizes and cross-sectional designs. Studies tended to be exploratory in design and many included a range of predictive and outcome variables rather than testing a specific association. The variation in the conceptualisations of social support in the studies included in this review, and in the tools used to measure it, reflect the variety observed in social support literature more generally [7]. This demonstrates that there is a lack of clarity about how best to define and operationalise social support, which may explain some of the inconsistent results in this review. Using global measures of support rather than measuring specific aspects risks failing to capture the ‘active ingredients’ of social support that may benefit mental health and psychological wellbeing after bereavement.

Additionally, many studies included samples that were predominantly female, over 30 years old and, where reported, of White ethnicity. This limited demographic variability, along with low response rates and convenience sampling through peer support groups, seem to be a feature of bereavement research in general [62, 64, 65] and limit the generalisability of results. The considerable variation in the potential confounding variables adjusted for in study models indicates inconsistency in what is thought to influence the relationship between social support and wellbeing. Key potentially confounding variables to account for in future analyses would include time [66] since bereavement, nature of relationship [67, 68] with the deceased, and pre-bereavement psychological wellbeing [69].

### Strengths and weaknesses of the review

The strengths of this review are that it used a systematic approach, including a thorough search of the grey literature. The lack of additional studies found through reference list searching, citation tracking and grey literature searching increases confidence that our search strategy was comprehensive and all relevant studies were retrieved. Although the majority of the title and abstract screening was completed by one author, we use independent rating of a proportion, and agreement between both reviewing authors was high.

Whilst it would be desirable to carry out a meta-analysis to produce a combined estimated effect size from the included studies, this was not possible in this review, given the differences in measurements of social support and the range of variables that each study controlled for in their statistical analysis models.

Some potentially relevant studies had to be excluded, as additional information about categorisation of deaths was not provided by authors: inclusion of these studies may have altered our main findings. It was also not possible to ensure completely consistent categorisation for the inclusion criteria used. Deaths through illness were excluded but can be sudden in certain circumstances (e.g. death caused by a heart attack), and some of the samples recruited through support groups may have completed measures of social support with reference to their support group rather than informal support from friends and family.

Overall, generalisability is limited by the homogeneity of included samples, but cross-cultural validity is relatively good for research in this area with the inclusion of minority and non-Western populations. The inclusion of samples recruited exclusively through support organisations would limit generalisability in these studies to those who have proactively sought help, and are well enough to be involved with these organisations.

The conclusions that can be drawn from this review are limited by the lack of published longitudinal studies to clarify the temporal direction of associations. The cross-sectional studies identified do not establish whether social support improves psychological wellbeing following bereavement, or if poor psychological wellbeing following bereavement reduces actual or perceived social support through its impact on relationships with others [70]. Establishing the temporal direction of associations is critical in understanding these relationships and using this in the development of interventions based on informal social support. Additionally, cross-sectional studies are unable to provide empirical evidence that supports or refutes either the main effects or the buffering model of social support as measuring the rate at which wellbeing improves according to level of social support received is necessary to distinguish between the two.

### Implications for research and practice

The findings of this research suggest that professionals supporting those who have been bereaved through sudden and/or violent causes, and especially those bereaved through suicide, should consider how the quantity and quality of available informal social support could be increased as a potential means to improve outcomes for their service users [20].

Priorities for research in this area should be to establish which specific types of informal support are most likely to improve psychological wellbeing, the temporal association between the degree of informal social support and a broad range of psychological wellbeing outcomes after bereavement, and the extent to which the degree of psychological morbidity influences the amount of social support available. The wider social support literature includes evidence to support a bidirectional relationship between social support and PTSD [71, 72]. Whilst general studies of support find that depression erodes social support [6, 73], very few studies have examined whether social support decreases the severity of depression [74]. Very little research has explored the relationship between CG and social support, most of which relates to sudden and/or violent losses, and so there is limited evidence of a relationship beyond this review. Cognitive models that explain CG highlight rumination as being a contributor to CG [75]. During the bereavement process, emotional support from others is likely to consist of opportunities to discuss the loss and its consequences, thus encouraging rumination [76]. This may explain the mixed evidence for an effect of social support on CG symptoms, as support overall is likely to improve wellbeing, but emotional support may exacerbate CG symptoms.

Given the inconsistencies in quantitative conceptualisation of the measurement of social support, qualitative research would complement this body of research by providing valuable insights to the bereavement experience in social settings. Qualitative work would also help identify the mechanisms by which some forms of informal social support may impact wellbeing after a loss.

## Conclusions

This systematic review of studies describing the relationship between post-loss informal social support and psychological wellbeing after sudden and/or violent bereavement suggests that informal social support may be important in improving psychological wellbeing following violent and/or sudden bereavement. However, current evidence is of insufficient quality or quantity to permit robust conclusions. Large, longitudinal studies with demographically varied samples are required to better understand the temporal direction of the relationships between different types of informal social support and psychological wellbeing following sudden bereavement. This information is important to the development and evaluation of programmes to enhance the availability or use of specific types of informal social support for people experiencing sudden and/or violent bereavement.

## Supplementary information

**Additional file 1 Appendix 1**. Search Strategy. **Appendix 2**. PRISMA checklist. **Appendix 3**. Data extraction.

## Data Availability

All citations identified are in the public domain. The datasets used during the current study are available from the corresponding author on reasonable request.

## Abbreviations

ASSIS: Arizona social support interview schedule
CG: Complicated grief
CISS: Coppel index of social support
CSS: Crisis support scale
FSSQ: Duke-UNC functional social support questionnaire
IES: Impact of event scale
ISS: Inventory of social support
MSPSS: Multidimensional scale of perceived social support
NOS: Newcastle-Ottawa Scale
NSSQ: Norbeck Social Support Questionnaire
PPI: Public and patient involvement
PSRS: Provisions of social relations scale
PSSQ: Perceived social support questionnaire
PTSD: Post-traumatic stress disorder
SSRS: Social support rating scale
