# Supplementary material for: A systematic review of studies describing the influence of informal social support on psychological wellbeing in people bereaved by sudden or violent causes of death
Source: BMC Psychiatry. 2020 May 29;20:265. doi: 10.1186/s12888-020-02639-4 (PMC7257446; doi:10.1186/s12888-020-02639-4)
Supplement: Supplementary file 1 — Additional file 1 Appendix 1. Search Strategy. Appendix 2. PRISMA checklist. Appendix 3. Data extraction. [file 12888_2020_2639_MOESM1_ESM.docx]

Supplementary files

Appendix 1: Search Strategy

**MEDLINE**

1. bereavement/
2. grief/
3. (bereave* or grief or griev* or mourn*)
4. widowhood/
5. (widow* or suicide survivor* or suicide loss*)
6. 1 or 2 or 3 or 4 or 5
7. Social support/
8. Social environment/
9. Social adjustment/
10. Financial support/
11. (practical support* or financial support* or monetary support* or financial gift* or monetary gift*)
12. (emotional support* or psychological support*)
13. (peer support* or informal support* or online support*)
14. (social support* or social adjustment* or social environment* or social network* or support system*)
15. Family/
16. Friends/
17. (friend* or companion* or relative* or famil*)
18. 7 or 8 or 9 or 10 or 11 or 12 or 13 or 14 or 15 or 16 or 17
19. quality of life/
20. (well being or wellbeing or well-bring or quality of life or life satisfaction)
21. mental disorders/
22. exp mental disorders/
23. mental health/
24. exp mental health/
25. (complicated grief or complicated grieving or prolonged grief or prolonged grieving)
26. Suicidal ideation/
27. Suicide, attempted/
28. (suicidal ideation or suicide attempt* or suicidal thought*)
29. Social isolation/
30. Social stigma/
31. Loneliness/
32. (isolat* or stigma* or loneliness or lonely)
33. 19 or 20 or 21 or 22 or 23 or 24 or 25 or 26 or 27 or 28 or 29 or 30 or 30 or 31 or 32
34. 6 and 18 and 33

**Cochrane**

"emotional support*" or "psychological support*" or friend* or companion* or relative* or famil* in Title Abstract Keyword OR "peer support*" or "informal support*" or "online support*" in All Text OR "social support*" or "social adjustment*" or "social environment*" or "social network*" or "support system*" in All Text OR family in Keyword OR friends in Keyword

**CINAHL**

TX ( “practical support*” or “financial support*” or “monetary support*” or “financial gift*” or “monetary gift*” or “emotional support*” or “psychological support*” or “peer support*” or “informal support*” or “online support*” or “social support*” or “social adjustment*” or “social environment*” or “social network*” or “support system*” or friend* or companion* or relative* or famil* ) AND TX ( bereave* or grief or griev* or mourn* or widow* or “suicide survivor*” or “suicide loss*” ) AND TX ( “quality of life” or well being or wellbeing or well-being or “life satisfaction” or “mental disorders” or “mental health” or complicated grief or “complicated grieving” or “prolonged grief” or “prolonged grieving” or “suicidal ideation” or “suicide attempt*” or isolat* or stigma* or loneliness or lonely )

**IBSS**

((bereave* OR grief OR griev* OR mourn* OR widow* OR "suicide survivor*" OR "suicide loss*") AND ("social support" OR "social environment" OR "social adjustment" OR "financial support" OR "practical support" OR "monetary support*" OR "financial gift" OR "monetary gift" OR "emotional support" OR "psychological support" OR "peer support" OR "informal support" OR "online support" OR "social adjustment" OR "social network" OR "support system" OR famil* OR friend* companion* OR relative*) AND ("quality of life" OR "well being" OR well-being OR wellbeing OR "life satisfaction" OR "mental disorders" OR "mental health" OR "complicated grief" OR "complicated grieving" OR "prolonged grief" OR "prolonged grieving" OR "suicidal ideation" OR "suicidal thought*" OR "suicide attempt*" OR "attempted suicide" OR isolat* OR stigma* OR loneliness OR lonely)) AND peer(yes) AND rtype.exact("Clinical Trial" OR "Clinical Trial, Phase I" OR "Controlled Clinical Trial" OR "Abstract" OR "Clinical Trial, Phase II" OR "Article" OR "Articles") AND stype.exact("Scholarly Journals")

**Psychinfo**

1.(TS= (bereave* OR grief OR griev* OR mourn* OR widow* OR "suicide survivor*" OR "suicide loss*")) *AND***DOCUMENT TYPES:** (Article)

2. (TS= ("quality of life" OR "well being" OR wellbeing OR well-being OR "life satisfaction" OR "mental disorder*" OR "mental health" OR "complicated grief" OR "complicated grieving" OR "prolonged grief" OR "prolonged grieving" OR "suicidal ideation" OR "attempted suicide" OR "suicide attempt" OR "suicidal thought*" OR loneliness OR lonely OR isolat* OR stigma*)) *AND***DOCUMENT TYPES:** (Article) 
3. (TS=("social support*" OR "social adjustment" OR "social environment*" OR "financial support*" OR "practical support*" OR "monetary support*" OR "financial gift*" OR "monetary gift*" OR "emotional support*" OR "psychological support*" OR "peer support*" OR "informal support*" OR "online support*" OR "social network*" OR "support system*" OR famil* OR friend* OR comparison* OR relative*)) *AND***DOCUMENT TYPES:** (Article) 
4. 1 AND 2 AND 3

Appendix 2: PRISMA checklist

| **Section/topic** | **#** | **Checklist item** | **Reported on page #** |
| --- | --- | --- | --- |
| **TITLE** | | |  |
| Title | 1 | Identify the report as a systematic review, meta-analysis, or both. | 1 |
| **ABSTRACT** | | |  |
| Structured summary | 2 | Provide a structured summary including, as applicable: background; objectives; data sources; study eligibility criteria, participants, and interventions; study appraisal and synthesis methods; results; limitations; conclusions and implications of key findings; systematic review registration number. | Under all sections, abstract |
| **INTRODUCTION** | | |  |
| Rationale | 3 | Describe the rationale for the review in the context of what is already known. | Paragraphs 4-5, introduction |
| Objectives | 4 | Provide an explicit statement of questions being addressed with reference to participants, interventions, comparisons, outcomes, and study design (PICOS). | Paragraph 6, introduction |
| **METHODS** | | |  |
| Protocol and registration | 5 | Indicate if a review protocol exists, if and where it can be accessed (e.g., Web address), and, if available, provide registration information including registration number. | Under “study selection”, methods |
| Eligibility criteria | 6 | Specify study characteristics (e.g., PICOS, length of follow-up) and report characteristics (e.g., years considered, language, publication status) used as criteria for eligibility, giving rationale. | Under “study inclusion” and “study selection”, methods |
| Information sources | 7 | Describe all information sources (e.g., databases with dates of coverage, contact with study authors to identify additional studies) in the search and date last searched. | Under “study selection”, methods |
| Search | 8 | Present full electronic search strategy for at least one database, including any limits used, such that it could be repeated. | Appendix 1 |
| Study selection | 9 | State the process for selecting studies (i.e., screening, eligibility, included in systematic review, and, if applicable, included in the meta-analysis). | Under “study selection”, methods |
| Data collection process | 10 | Describe method of data extraction from reports (e.g., piloted forms, independently, in duplicate) and any processes for obtaining and confirming data from investigators. | Under “study selection” and “data extraction”, methods |
| Data items | 11 | List and define all variables for which data were sought (e.g., PICOS, funding sources) and any assumptions and simplifications made. | Appendix 3 |
| Risk of bias in individual studies | 12 | Describe methods used for assessing risk of bias of individual studies (including specification of whether this was done at the study or outcome level), and how this information is to be used in any data synthesis. | Under “quality appraisal”, methods |
| Summary measures | 13 | State the principal summary measures (e.g., risk ratio, difference in means). | - |
| Synthesis of results | 14 | Describe the methods of handling data and combining results of studies, if done, including measures of consistency (e.g., I^2^) for each meta-analysis. | Under “summary of findings”, methods |

Appendix 3: data extraction

Reference

Title

Year

Country

Study type

Sample size

Sample demographics

Inclusion/exclusion criteria

Period of loss

Type of death

Social support measurement

Reference for social support validation

Type of social support measured

Length of follow-up

Analysis model used

Results

Evidence to support hypothesis (yes/no)

Reported limitations

Reported strengths

Limitations

Strengths

.
